# Local and Non-local Effects of Foam Rolling on Passive Soft Tissue Properties and Spinal Excitability

**DOI:** 10.3389/fphys.2021.702042

**Published:** 2021-06-25

**Authors:** Masatoshi Nakamura, Andreas Konrad, Ryosuke Kiyono, Shigeru Sato, Kaoru Yahata, Riku Yoshida, Koki Yasaka, Yuta Murakami, Futaba Sanuki, Jan Wilke

**Affiliations:** ^1^Institute for Human Movement and Medical Sciences, Niigata University of Health and Welfare, Niigata, Japan; ^2^Department of Physical Therapy, Niigata University of Health and Welfare, Niigata, Japan; ^3^Institute of Human Movement Science, Sport and Health, University of Graz, Graz, Austria; ^4^Department of Sports Medicine and Exercise Physiology, Institute of Occupational, Social and Environmental Medicine, Goethe University Frankfurt, Frankfurt, Germany

**Keywords:** shear elastic modulus, dorsiflexion range of motion, roller massage, cross-transfer effect, H/M ratio

## Abstract

In sports and clinical settings, roller massage (RM) interventions are used to acutely increase range of motion (ROM); however, the underlying mechanisms are unclear. Apart from changes in soft tissue properties (i.e., reduced passive stiffness), neurophysiological alterations such as decreased spinal excitability have been described. However, to date, no study has investigated both jointly. The purpose of this trial was to examine RM’s effects on neurophysiological markers and passive tissue properties of the plantar flexors in the treated (ROLL) and non-treated (NO-ROLL) leg. Fifteen healthy individuals (23 ± 3 years, eight females) performed three unilateral 60-s bouts of calf RM. This procedure was repeated four times on separate days to allow independent assessments of the following outcomes without reciprocal interactions: dorsiflexion ROM, passive torque during passive dorsiflexion, shear elastic modulus of the medial gastrocnemius muscle, and spinal excitability. Following RM, dorsiflexion ROM increased in both ROLL (+19.7%) and NO-ROLL (+13.9%). Similarly, also passive torque at dorsiflexion ROM increased in ROLL (+15.0%) and NO-ROLL (+15.2%). However, there were no significant changes in shear elastic modulus and spinal excitability (*p* > 0.05). Moreover, significant correlations were observed between the changes in DF ROM and passive torque at DF ROM in both ROLL and NO-ROLL. Changes in ROM after RM appear to be the result of sensory changes (e.g., passive torque at DF ROM), affecting both rolled and non-rolled body regions. Thus, therapists and exercise professionals may consider applying remote treatments if local loading is contraindicated.

## Introduction

Improving flexibility is a frequent goal in sports. Besides representing a potential hallmark of athletic performance, it may also be related to injury risk. Previous studies reported an association of low flexibility (Witvrouw et al., 2001), high muscle stiffness (Pickering Rodriguez et al., 2017), and the occurrence of musculoskeletal complaints. Besides stretching and mobility exercise, rolling massage (RM) is another popular self-treatment to increase flexibility. In essence, it uses various tools such as polypropylene rollers (then called foam rolling), massagers, balls, and other similar devices to apply a compressive force to the soft tissue of the targeted body region.

Previous studies (Krause et al., 2019; Wilke et al., 2019, 2020; Behm et al., 2020) have not only shown an immediate increase in range of motion (ROM) following foam rolling but also revealed the absence of changes in athletic performance (Macdonald et al., 2013; Behara and Jacobson, 2017). This is relevant because static stretching for more than 60 s can likely decrease muscle strength and athletic performance if performed isometrically (Kay and Blazevich, 2012; Behm et al., 2016, 2021). Although foam rolling appears to be a useful technique for athletes, its mechanisms remain a matter of debate. Initially, foam rolling was assumed to modify local tissue characteristics and properties. Hotfiel et al. (2017) found an increase in tissue perfusion, which in turn could cause a decrease in mechanical stiffness. Moreover, Morales-Artacho et al. (2017) and Wilke et al. (2019) showed decreased mechanical stiffness immediately after foam rolling. However, previous studies investigated the immediate changes in muscle hardness or muscle stiffness after foam rolling, showing increased ROM but no significant differences in the aforementioned morphological outcomes (Yoshimura et al., 2020; Nakamura et al., 2021a). As a consequence, the initial assumptions that RM may primarily cause alterations of the mechanical soft tissue properties have been questioned (Behm and Wilke, 2019).

Considering the mixed evidence regarding local foam rolling effects, recent research has increasingly focused on neurophysiological changes. For instance, a previous study investigated the effect of rolling intervention on spinal excitability (Young et al., 2018) and corticospinal excitability (Aboodarda et al., 2018). With regard to functional outcomes, it was shown that foam rolling also induces changes in non-treated areas such as an increased ROM of the contralateral leg, which potentially reflects systemic changes in stretch sensation (i.e., stretch tolerance) (Kelly and Beardsley, 2016; Killen et al., 2019). Yet, contrarily, another previous study showed no significant increase in ROM of the contralateral leg (Grabow et al., 2017). Besides ROM, other studies examined the pressure pain threshold (PPT). Interestingly, some reported a non-local increase in PPT, again suggesting that neural changes could be responsible for flexibility changes after foam rolling (Aboodarda et al., 2015; Cavanaugh et al., 2017).

Summarizing the evidence regarding foam rolling mechanisms in a topical review, Behm and Wilke (2019) pointed out that the relative contributions of structural (e.g., passive stiffness) and neural effects (e.g., spinal excitability; i.e., H/M ratio) are yet to be elucidated. However, to our knowledge, no other study has examined the surrogates of both in one study. Examining structural and neural markers in the same participants and both the treated and non-treated body regions could help to understand better how foam rolling increases ROM.

Against this background, this study was geared to (1) investigate the effects of roller massage (RM) on passive tissue properties and neurological changes as well as their relationship with ROM in the treated leg and (2) investigate the possible cross-education effect of the passive properties and neurological parameters.

## Materials and Methods

### Experimental Design

**FIGURE 1:**
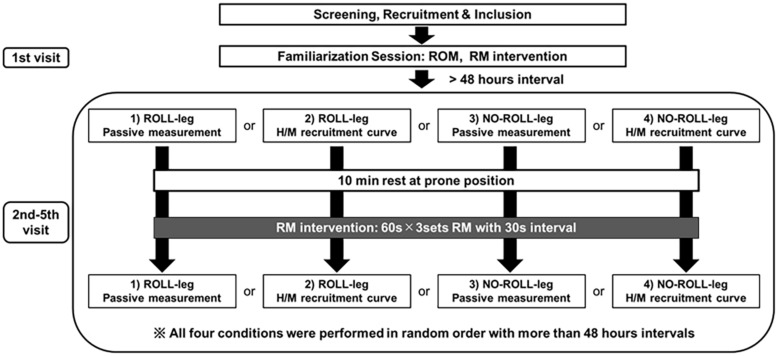
Flowchart of the study. The participants were applied roller massage (RM) intervention in the dominant leg, and we defined the treated leg as ROLL, and the non-treated leg as NO-ROLL, respectively. The outcome variables consisted of two trials, and one trial was composed of passive tissue property measurements (dorsiflexion range of motion [DF ROM], passive torque, and shear elastic modulus) and pressure pain threshold, and the other trial was composed of spinal excitability (H/M recruitment curve) measurement. Four measurements (2 sections × 2 sides [ROLL and NO-ROLL]) were conducted in random order with more than a 48-h interval.

A randomized, repeated-measures experimental design was used to investigate the neural and tissue-specific effects of RM. The participants, in random order, visited the laboratory five times (Figure 1). The first day was a familiarization session, including a demonstration of the RM intervention and the measurement protocols. The remaining four sessions included four measurement trials with an identical intervention (3 × 60-s RM on the calf). This approach with repeated interventions was chosen in order to prevent reciprocal interactions between the various measurements. The participants have been applied RM intervention in the dominant leg (prefer to kick the ball), and we defined the treated leg as ROLL, and the non-treated leg as NO-ROLL. The outcome variables consisted of two measurement trials, e.g., one trial was composed of passive tissue property measurements (DF ROM, passive torque, and shear elastic modulus) and PPT, and the other trial was composed of H/M recruitment curve (spinal excitability) measurement. Four measurements (2 trials [passive property and spinal excitability] × 2 sides [ROLL and NO-ROLL]) were conducted in random order with more than a 48-h interval.

The protocol for the four measurement days was identical and based on a previous study (Young et al., 2018). After the baseline measurement preceding the RM intervention (PRE), a 10-min break was given, and after the break, three sets of RM intervention for 60 s were performed. At 2 min after RM intervention, either assessment of the passive properties or spinal excitability (POST) measurement was performed.

### Participants

Fifteen healthy adults volunteered to participate in this crossover study (mean ± SD: age, 22.8 ± 3.0 years; height, 165.2 ± 7.4 cm; weight, 58.7 ± 8.0 kg; male: female, 7: 8) Individuals with a history of neuromuscular disease and musculoskeletal injury involving the lower extremities were excluded. They were fully informed about the procedures and purpose of the study and provided written informed consent. The Ethics Committee of the Niigata University of Health and Welfare, Niigata, Japan (Procedure #18304), approved the study, which complied with the Declaration of Helsinki.

G^∗^Power (v 3.0.10; Dr. Franz Faul, Kiel University, Kiel, Germany) was used to perform an *a priori* sample-size calculation (primary outcome: ROM) for a repeated-measure analysis of variance (ANOVA) (effect size = 0.4, α = 0.05, and power = 0.80) based on a previous study (Phillips et al., 2018). It yielded a minimum of *n* = 15 individuals to be recruited for this study.

### RM Intervention

Roller massage was applied using a roller massager (TheraBand, Akron, OH, United States). Participants were in a prone position on a treatment bed. An examiner moved the roller massager forward and backward, rolling on the muscle belly of the gastrocnemius. Speed was controlled with a metronome set to 60 bpm (Smart Metronome; Tomohiro Ihara, Japan). At each beep, the examiner foam moved the massager one stroke up or down. The intensity was steered based on the participants’ feedback, targeting a 7/10 in discomfort on a numerical rating scale (0, no discomfort; 10, maximal discomfort) (Smith et al., 2019; Kiyono et al., 2020a). Based on previous studies, participants performed three sets of RM for 60 s with a 30-s break between sets (Smith et al., 2019; Behm et al., 2020; Kiyono et al., 2020a).

### Assessment of the DF ROM and Passive Torque

The participants were in the prone position with a 0° knee angle (i.e., the anatomical position), having the foot of the measured leg attached to the footplate of a dynamometer. Using the passive mode, the device moved the ankle into passive dorsiflexion at a speed of 5°/s, starting from 30° plantar flexion (i.e., anatomical neutral position was 0°) (Kiyono et al., 2020a; Nakamura et al., 2021b). Measurements were ended when the participants experienced discomfort and stopped the dynamometer by activating the safety trigger (Kiyono et al., 2020a; Nakamura et al., 2021b). The achieved angle at this point was documented as the DF ROM. The data collected during the ROM measurement was also used to calculate the passive–resistive torque of the ankle joint, which represents a measure of dynamic stiffness. In this study, dynamic stiffness was calculated as the change in the passive torque from the neutral ankle position (0°) to the least dorsiflexion angle before and after RM intervention divided by the change of the joint angle (Konrad et al., 2017). Additionally, passive torque at DF ROM was defined as the sensory perception index (Morse et al., 2008; Nakamura et al., 2018). DF ROM and passive torque were measured twice, utilizing its average value for further analysis.

### Assessment of the Shear Elastic Modulus

Shear elastic modulus of MG was measured by means of an ultrasonic shear wave elastography (SWE) imaging device (Aixplorer SuperSonic Imagine, Aix-en-Provence, France) with a linear probe (4–15 MHz, SuperLinear 15-4, Vermon, Tours, France) and using the SWE mode (musculoskeletal preset, penetration mode, smoothing level 5, persistence medium). SWE provides a two-dimensional map of the shear elastic modulus on the B-mode image of the target tissue at 1 Hz with a spatial resolution of 1 × 1 mm (Lacourpaille et al., 2012; Umehara et al., 2021). Measurements were obtained at a neutral ankle position and 20° plantar flexion, which is similar to the positions of DF ROM measurements. We measured the shear elastic modulus at these angles to evaluate the changes in the passive mechanical property of the muscle under tension (neutral position) and without tension (20° plantar flexion). Also, the H and M waves were measured at 20° plantar flexion (described below), and we investigated the changes in the passive mechanical properties at that angle. The shear elastic modulus of MG was measured at 30% of the lower leg length from the popliteal crease to the lateral malleolus near the point of the lower leg maximal cross-sectional area (Nakamura et al., 2019; Kiyono et al., 2020a,b; Sato et al., 2020). The size of the region of interest was 10 × 20 mm^2^ near the center of MG, with a 5-mm-diameter-circle analysis area at the center of the region (Saeki et al., 2019). Elastographic images of the MG long axis were obtained twice. Based on previous studies (Nakamura et al., 2019; Fukaya et al., 2020; Sato et al., 2020), the shear elastic modulus was calculated by dividing the obtained Young’s modulus by 3, whereas the ultrasound measurements were measured twice in each ankle position. The average shear elastic modulus value was used for analysis.

### Pressure Pain Threshold

For PPT measurements, we employed an algometer (NEUTONE TAM-22 (BT10); TRY ALL, Chiba, Japan). Participants adopted a prone position on the treatment bed similar to that for the DF ROM measurements. The joint position was 20° at the plantar flexion position. With continuously increasing pressure, the metal rod of the algometer was used to compress the soft tissue in the measurement area. Participants were instructed to immediately press a trigger when pain was experienced rather than just pressure. The value read from the device at this time point (kilograms per square centimeter) corresponded to the PPT. Based on previous studies (Kim and Lee, 2018; Naderi et al., 2020), the mean value (kilograms per square centimeter) of three repeated measurements was taken with a 30-s interval for data analysis.

### Electromyography

Electromyographic activity of the soleus muscle was recorded using self-adhesive electrodes with recording surface electrodes (Ambu, Blue Sensor N). Before measurements, the skin was cleaned with alcohol to improve conductivity. Electrodes were placed on the soleus according to SENIAN (Hermens et al., 2000), with the ground electrode between the electrical stimulation and surface electromyogram electrodes. Electromyographic activity was filtered with a 10–1,000-Hz band-pass filter (Fa-DL-720-140, 4Assist, Tokyo, Japan) and digitally stored (10 kHz sampling rate) on a personal computer for offline analysis, which was performed using PowerLab 8/30 (AD Instruments, Colorado Springs, CO, United States) and LabChart 7 (AD Instruments). In the passive dorsiflexion test, the soleus muscle activity was monitored to confirm whether the participant was relaxed, ensuring no high EMG activity occurrence.

### H-Reflex, M-Wave

In the spinal cord mononeuron excitability assessment, participants were instructed to lie in a prone position on the treatment bed with the knee at a 0° knee angle and the ankle in a 20° plantar flexion position. The participants were requested to relax completely, which we ensured by controlling EMG activity. In this study, electrical stimulations were delivered using a constant current stimulator (Isolator SS-104 J: Nihon Kohden Corporation) targeting the tibial nerve using 1-ms pulses. Based on previous researches (Hirabayashi et al., 2018, 2020), the tibial nerve was selectively stimulated in a monopolar manner, inducing soleus H-reflex and M waves. The anode was placed on the patella and the cathode on the popliteal fossa overlying the nerve at a position using the electrode (Blue Sensor N, size: 30 × 22 mm), providing the greatest H wave amplitude at the smallest stimulus intensity, which was identified by stimulating the different skin surface sites with relatively low currents. Electrodes were attached to the skin using surgical tape to prevent unwanted movement during testing.

H/M recruitment curves were assessed before and after sessions. At the beginning of each, H-reflex and M wave recruitment curves were measured under resting conditions to determine the H-reflex (H_*max*_) and M wave (M_*max*_) maximum amplitudes. Stimulations were increased by 0.5 mA every 10 s until M_*max*_ was reached, with the corresponding intensity for all stimulations retained for analysis. The three stable H_*max*_ and M_*max*_ are measured, and the average value of each is used for analysis. The H/M ratio, an index of spinal excitability, was calculated from the measured H_*max*_ and M_*max*_ (Blazevich et al., 2012, 2014; Stutzig and Siebert, 2017).

### Pilot Measurements of Test–Retest Reliability

The test–retest reliability of the measurements (all variables) was determined by the coefficient variation (CV) and intraclass correlation coefficient (ICC) using eight healthy students (four men, four women, 21.9 ± 1.1 years, 162.9 ± 7.3 cm, 55.2 ± 9.0 kg), with a 1-week washout between the two measures. The CV of and passive torque at DF ROM, PPT, shear elastic modulus at 0° and 20°, and H/M ratio were 9.9 ± 13.3, 6.4 ± 3.9, 4.0 ± 3.3, 2.0 ± 1.4, 3.6 ± 2.4, and 9.7 ± 8.7%, respectively, and the ICCs for the measurements were 0.96, 0.96, 0.97, 0.92, 0.91, 0.88, and 0.96, respectively.

### Statistical Analysis

Normal distribution of data was assessed with the Shapiro–Wilk test. Examinations showed that no violations of the testing assumption were present. Comparisons of the parameters’ PRE values were made between the ROLL and NO-ROLL legs using the paired *t*-test. If a significant difference was noted between PRE values, we used a paired *t*-test with Bonferroni corrections to compare the significant differences between the PRE and POST values of each leg, and the Wilcoxon signed-rank test was used to compare the pre-post changes (in percentage) between the ROLL and NO-ROLL leg. Conversely, if no significant difference was noted between PRE values, a two-way repeated-measure ANOVA (time [PRE vs. POST] × legs [ROLL leg vs. NO-ROLL leg]) was used to investigate the interactions and main effects in the experiment. If a main effect for time (without interaction) was noted, significant differences between the PRE and POST values were determined using a paired *t*-test. The effect size was calculated as a difference in the mean value between PRE and POST divided by the pooled standard deviation (SD) (Cohen, 1988) with effect sizes of 0.00–0.19, 0.20–0.49, 0.50–0.79, and ≥0.80 being considered trivial, small, moderate, and large, respectively. Finally, the correlations between changes in ROM and each variable using Spearman’s rank correlation coefficient were investigated. A 5% significance level was used. All results are shown as mean ± SD. The Statistical Package for the Social Sciences, version 24.0 (IBM Corp., Armonk, NY, United States) was used for statistical analyses.

## Results

### Comparison of Preintervention Values

All values are shown in Tables 1, 2. Paired *t*-tests showed a significant difference in PPT between the PRE values of the ROLL and NO-ROLL legs (*p* = 0.018; Table 2). No significant baseline differences were found concerning the other parameters (DF ROM, dynamic stiffness, passive torque at DF ROM, shear elastic modulus, and H/M ratio).

**TABLE 1:** Change in dorsiflexion range of motion (DF ROM), dynamic stiffness, passive torque at DF ROM, shear elastic modulus, and H/M ratio before (PRE) and after (POST) the roller massage intervention for treated side (ROLL) and non-treated side (NO-ROLL) legs.

		ROLL leg	NO-ROLL leg	Interaction effect
DF ROM (°)	PRE	29.0 ± 11.0	34.6 ± 5.8	*F* = 0.19, *P* = 0.669 η_*p*_^2^ = 0.01
	POST	34.7 ± 8.0	39.4 ± 6.6	
	Effect size	*d* = 0.61	d = 0.78	
Dynamic stiffness (Nm/°)	PRE	0.70 ± 0.24	0.75 ± 0.21	*F* = 1.047, *P* = 0.324 η_*p*_^2^ = 0.07
	POST	0.64 ± 0.22	0.73 ± 0.23	
	Effect size	*d* = 0.23	*d* = 0.05	
Passive torque at DF ROM (Nm)	PRE	24.7 ± 13.0	28.3 ± 10.7	*F* = 0.378, *P* = 0.55 η_*p*_^2^ = 0.026
	POST	28.4 ± 13.2	32.6 ± 12.7	
	Effect size	d = 0.28	d = 0.36	
Shear elastic modulus at neutral (kPa)	PRE	9.0 ± 1.0	9.5 ± 1.9	*F* = 0.43, *P* = 0.523 η_*p*_^2^ = 0.03
	POST	9.7 ± 1.6	9.9 ± 1.43	
	Effect size	*d* = 0.59	*d* = 0.25	
Shear elastic modulus at plantarflexion 20° (kPa)	PRE	4.9 ± 0.7	6.0 ± 2.2	*F* = 2.604, *P* = 0.129 η_*p*_^2^ = 0.157
	POST	5.3 ± 0.8	5.5 ± 1.3	
	Effect size	*d* = 0.52	*d* = 0.25	
H/M ratio	PRE	0.72 ± 0.26	0.69 ± 0.20	*F* = 0.271, *P* = 0.611 η_*p*_^2^ = 0.019
	POST	0.72 ± 0.33	0.67 ± 0.20	
	Effect size	*d* = 0.00	*d* = 0.07	

*Interaction effect for the comparison between the intervention and non-intervention sides based on two-way repeated measures on a split-plot analysis of variance on the right columns. Data presented as mean ± standard deviation.*

**TABLE 2:** Change in pressure pain threshold before (PRE) and after (POST) the roller massage intervention for treated side (ROLL) and non-treated side (NO-ROLL) legs.

		ROLL leg	NO-ROLL leg
Pressure pain threshold (kg)	PRE	3.06 ± 0.97	2.43 ± 0.81*
	POST	3.76 ± 1.25	2.95 ± 0.83
	Effect size	*d* = 0.63	*d* = 0.63
	Δ change (%)	22.2 ± 17.5	26.8 ± 36.1

Data presented as mean ± standard deviation. *p < 0.05; significantly different between ROLL and NO-ROLL leg.

### Pre–Post Changes in the ROLL and NO-ROLL Legs

The two-way repeated-measure analysis of variance did not show interaction effects (ROLL/NO-ROLL leg) in any other outcome (*p* > 0.05; Table 1). Main effects for time were found in DF ROM and passive torque at DF ROM (*p* < 0.05) but not for dynamic stiffness, shear elastic modulus, and H/M ratio (*p* > 0.05). According to *post hoc* testing with paired *t*-tests, DF ROM and PPT increased after RM (*p* < 0.01). In addition, regarding PPT, paired *t*-tests with Bonferroni corrections showed a significant increase after RM intervention in both ROLL and NO-ROLL sides, but the Wilcoxon signed-rank test revealed no significant difference in the relative pre–post changes between the ROLL and NO-ROLL leg (*p* = 0.609; Table 2).

### Relationship Between the Changes in DF ROM and All Variables

**FIGURE 2:**
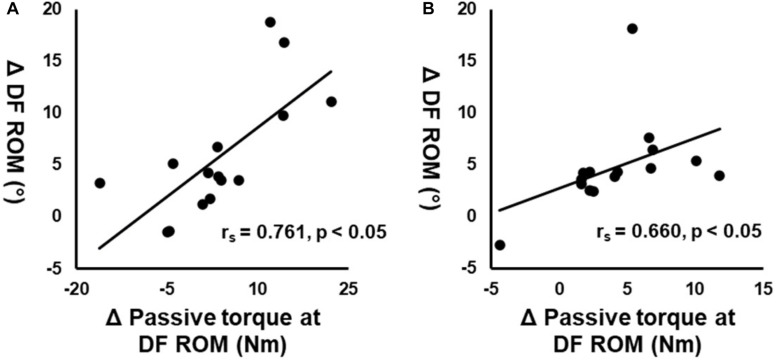
Correlation between the changes in dorsiflexion range of motion (DF ROM) before (PRE) and after roller massage (POST) and changes in passive torque at DF ROM on the treated **(A)** and non-treated **(B)** legs.

Significant correlations were observed between the changes in DF ROM and passive torque at DF ROM in both ROLL and NO-ROLL legs (Figure 2A: *r*_*s*_ = 0.761, *p* < 0.01; Figure 2B: *r*_*s*_ = 0.660, *p* < 0.01, respectively), whereas no significant associations occurred between changes in DF ROM and PPT (*r*_*s*_ = 0.311, *p* = 0.26; *r*_*s*_ = 0.048, *p* = 0.864), dynamic stiffness (*r*_*s*_ = 0.129, *p* = 0.65; *r*_*s*_ = –0.176, *p* = 0.53), shear elastic modulus at neutral (*r*_*s*_ = 0.418, *p* = 0.121; *r*_*s*_ = −0.021, *p* = 0.94), 20° plantarflexion (*r*_*s*_ = 0.325, *p* = 0.237; *r*_*s*_ = −0.15, *p* = 0.594), and H/M ratio (*r*_*s*_ = 0.136, *p* = 0.63; *r*_*s*_ = 0.239, *p* = 0.39) at both legs.

## Discussion

To the best of our knowledge, our study was the first to jointly investigate the acute effects of RM on structural and neuronal outcomes. On both sides, the results showed a significant increase in DF ROM and PPT but no significant changes in dynamic stiffness, shear elastic modulus, and spinal excitability. Since the changes in DF ROM correlated with changes in passive torque at DF ROM, the increasing ROM after the RM intervention could be associated with stretch tolerance changes on both treated and non-treated sides.

The ROM increases following RM in ROLL and NO-ROLL are generally in accordance with the findings of previous trials (Kelly and Beardsley, 2016; Killen et al., 2019). Beyond this, we found no significant interaction effect and no significant difference in the changes between legs. Similar to our findings, Kelly and Beardsley (2016) and Killen et al. (2019) reported no significant difference in the effects of foam rolling on ROM in the two legs. In sum, it appears that ROM increases after foam rolling locally and remotely at a comparable magnitude. This may mean that foam rolling-induced ROM changes largely stem from neural mechanisms, i.e., stretch tolerance, and to a minor degree by local/structural alterations, i.e., passive stiffness of the muscle–tendon unit or the skeletal muscles.

In addition to increased ROM, the present study found significant changes in PPT on both sides. Previous studies (Cheatham and Baker, 2017; Cheatham and Stull, 2019) showed similar results, which strengthens the assumption that foam rolling modifies sensory perception. Behm and Wilke (2019) proposed that the gate control theory, parasympathetic hyperactivity, and diffuse noxious inhibitory control could be involved. Previous studies suggested the involvement of the central pain modulatory system in contralateral PPT increase (Aboodarda et al., 2015; Cavanaugh et al., 2017). Although the exact mechanism in the present study is unknown, the aforementioned processes may also have caused the changes in PPT in our case.

Foam rolling and RM intervention have been shown to modify myofascial viscoelastic properties by mechanisms such as thixotropy (reduced viscosity), myofascial restriction reduction, fluid changes, and cellular responses (Kelly and Beardsley, 2016; Cheatham and Stull, 2019). No significant changes were found in dynamic stiffness and shear elastic modulus in both ROLL and NO-ROLL legs, respectively. This is of interest because Morales-Artacho et al. (2017) reported a significant decrease in hamstring muscle stiffness immediately after foam rolling. Contrarily, Yoshimura et al. (2020) showed no significant decrease in muscle hardness of the gastrocnemius after a 300-s foam rolling intervention. In addition, Nakamura et al. (2021a) showed that different duration foam rolling intervention (30, 90, and 300 s) could not cause changes in shear elastic modulus of the same muscle, which is consistent with the results of the present study (Nakamura et al., 2021a). Consequently, the different target muscles may respond individually to foam rolling intervention, and the mechanical effects of foam rolling seem to require further investigation.

For the ROM change noted after the RM intervention, Grabow et al. (2018) suggested that ROM increases are related to a spinal excitability change. A previous study (Young et al., 2018) investigated RM effects on the H-reflex and reported a decrease during RM, with transient effects and an immediate return to baseline after the intervention. However, our study showed no significant change in the H/M ratio. The difference between the trial of Young and colleagues and ours’ may be explained by the timing of measurements. The previous study (Young et al., 2018) measured the H-reflex during and immediately after the RM intervention, whereas the current study examined it at 2 min post-intervention. Therefore, RM-induced changes of spinal excitability may occur during the intervention but with effects that return immediately to baseline after the intervention.

Our correlation analysis showed a significant association between the changes in DF ROM and the passive torque at DF ROM in both ROLL and NO-ROLL (Figures 2A,B) but not in other variables. These results suggested that the DF ROM increase on both sides after RM was related to changes in stretch perception, i.e., stretch tolerance. According to previous studies, moderate-to-strong correlations between ROM and passive torque changes at ROM were indicated as altered type III or IV afferent activity, influencing pain perception and the magnitude of ROM changes (Magnusson et al., 1996; Weppler and Magnusson, 2010). In addition, this association could be good evidence for the importance of neurological adaptation (i.e., change in stretch tolerance) for the acute increase in ROM (Kay et al., 2015). Killen et al. (2019) found that foam rolling intervention increased ROM on the non-intervention side, suggesting that stretch tolerance was involved in the increase in ROM. Moreover, Aboodarda et al. (2015) showed that PPT was increased after a foam rolling intervention on both the intervention side and the non-intervention side. Thus, this suggested that the central nervous system changes; for example, the noxious counter-irritating mechanism is responsible for increasing PPT on the non-intervention side after the foam rolling intervention (Aboodarda et al., 2015). The details of the mechanism of stretch tolerance change in the non-intervention side (NO-ROLL) are unclear in this study, but it is possible that the central nervous system could be responsible for stretch tolerance change and the increase in ROM on the non-intervention side. However, Behm and Wilke (2019) suggested that PPT change is related to the increase in ROM after foam rolling intervention. Although the present study results showed increased PPT on both legs, no significant correlations were observed between the changes in DF ROM and PPT. The difference between the changes in PPT and stretch tolerance is unclear. Also, PPT measurement assessed not deep tissue but superficial tissue, and therefore might not assess RM-induced muscle changes. The present study suggested that PPT changes are not related to ROM changes.

Other factors involved in the ROM change could be changes in muscle stiffness and/or spinal excitability. However, the current study showed that RM intervention increased ROM, with no changes in shear elastic modulus and H/M ratio, i.e., mechanical property and neurological change, suggesting that muscle stiffness and H/M ratio changes were not related to DF ROM increase in both legs after the RM intervention. Young et al. (2018) showed decreased H-reflex during RM intervention, which returned to baseline after RM intervention. In addition, the results of this study showed no significant changes in H/M ratio and no significant correlations between the changes in DF ROM and H/M ratio on both legs. Thus, the present study suggested that muscle stiffness and H/M ratio changes were not related to the increase in ROM after RM intervention.

This study had several limitations. In the previous study (Young et al., 2018), H-reflex was measured during the RM intervention. In contrast, in this study, as indicated, the H/M ratio was measured only 2 min after the RM intervention. Future studies may consider investigating the effects of RM interventions on H/M ratio and muscle stiffness during RM application. Also, we did not measure the control condition (without RM intervention). In addition, because the different measurements may mutually influence each other, we had to repeat the treatment four times on separate days. Thus, although we used the exact same protocol in the same participants in one study, the different outcomes, technically, have not been assessed simultaneously.

## Conclusion

This study investigated the effect of RM on DF ROM, passive torque, shear elastic modulus, PPT, and H/M ratio on both the intervention and non-intervention sides. The results showed that DF ROM and PPT increased after treatment in both legs. The increases in ROM were related to the changes in stretch tolerance but not muscle stiffness, PPT, and spinal excitability. Interestingly, this study showed the same degree of RM intervention effect on DF ROM and the intervention and non-intervention sides. These results could be a treatment option in clinical and sports settings. Specifically, unilateral RM intervention on the healthy side may maintain or increase the ROM of the disabled side even if RM intervention could not be adaptable owing to injury or pain.

## Data Availability Statement

The raw data supporting the conclusions of this article will be made available by the authors, without undue reservation.

## Ethics Statement

The studies involving human participants were reviewed and approved by this study was approved by the Ethics Committee of the Niigata University of Health and Welfare, Niigata, Japan (Procedure #18304), and has complied with the Declaration of Helsinki requirements. The patients/participants provided their written informed consent to participate in this study.

## Author Contributions

MN contributed to the study design and data collection, and drafted and critical revision to the manuscript. RK, SS, KaY, RY, KoY, YM, and FS contributed to the data collection and made critical revisions to the manuscript. AK and JW contributed to the study design and data analysis and made critical revisions to the manuscript. All the authors approved the final version of the manuscript and agreed to be accountable for all aspects of the work.

## Conflict of Interest

The authors declare that the research was conducted in the absence of any commercial or financial relationships that could be construed as a potential conflict of interest.

## References

[B1] Aboodarda S. J. Greene R. M. Philpott D. T. Jaswal R. S. Millet G. Y. Behm D. G. (2018). The effect of rolling massage on the excitability of the corticospinal pathway. *Appl. Physiol. Nutr. Metab.* 43 317–323. 10.1139/apnm-2017-0408 29084391

[B2] Aboodarda S. J. Spence A. J. Button D. C. (2015). Pain pressure threshold of a muscle tender spot increases following local and non-local rolling massage. *BMC Musculoskelet Disord.* 16:265. 10.1186/s12891-015-0729-5 26416265 PMC4587678

[B3] Behara B. Jacobson B. H. (2017). Acute effects of deep tissue foam rolling and dynamic stretching on muscular strength, power, and flexibility in division I Linemen. *J. Strength Cond. Res.* 31 888–892. 10.1519/JSC.0000000000001051 26121431

[B4] Behm D. G. Alizadeh S. Hadjizadeh Anvar S. Mahmoud M. M. I. Ramsay E. Hanlon C. (2020). Foam rolling prescription: A mentary. *J. Strength Cond. Res.* 34 3301–3308. 10.1519/JSC.0000000000003765 33105383

[B5] Behm D. G. Blazevich A. J. Kay A. D. Mchugh M. (2016). Acute effects of muscle stretching on physical performance, range of motion, and injury incidence in healthy active individuals: a systematic review. *Appl. Physiol. Nutr. Metab.* 41 1–11. 10.1139/apnm-2015-0235 26642915

[B6] Behm D. G. Kay A. D. Trajano G. S. Blazevich A. J. (2021). Mechanisms underlying performance impairments following prolonged static stretching without a comprehensive warm-up. *Eur. J. Appl. Physiol.* 121 67–94. 10.1007/s00421-020-04538-8 33175242

[B7] Behm D. G. Wilke J. (2019). Do self-myofascial release devices release Myofascia? rolling mechanisms: a narrative review. *Sports Med.* 49 1173–1181. 10.1007/s40279-019-01149-y 31256353

[B8] Blazevich A. J. Cannavan D. Waugh C. M. Miller S. C. Thorlund J. B. Aagaard P. (2014). Range of motion, neuromechanical, and architectural adaptations to plantar flexor stretch training in humans. *J. Appl. Physiol.* 117 452–462. 10.1152/japplphysiol.00204.2014 24947023

[B9] Blazevich A. J. Kay A. D. Waugh C. Fath F. Miller S. Cannavan D. (2012). Plantarflexor stretch training increases reciprocal inhibition measured during voluntary dorsiflexion. *J. Neurophysiol.* 107 250–256. 10.1152/jn.00407.2011 21975448

[B10] Cavanaugh M. T. Döweling A. Young J. D. Quigley P. J. Hodgson D. D. Whitten J. H. (2017). An acute session of roller massage prolongs voluntary torque development and diminishes evoked pain. *Eur. J. Appl. Physiol.* 117 109–117. 10.1007/s00421-016-3503-y 27853885

[B11] Cheatham S. W. Baker R. (2017). Differences in pressure pain threshold among men and women after foam rolling. *J. Bodyw. Mov. Ther.* 21 978–982. 10.1016/j.jbmt.2017.06.006 29037655

[B12] Cheatham S. W. Stull K. R. (2019). Roller massage: Comparison of three different surface type pattern foam rollers on passive knee range of motion and pain perception. *J. Bodyw. Mov. Ther.* 23 555–560. 10.1016/j.jbmt.2019.05.002 31563369

[B13] Cohen J. (ed). (1988). *Statistical Power Analysis for the Behavioral Sciences.* Routledge: Hillsdale.

[B14] Fukaya T. Nakamura M. Sato S. Kiyono R. Yahata K. Inaba K. (2020). The relationship between stretching intensity and changes in passive properties of gastrocnemius muscle-tendon unit after static stretching. *Sports (Basel)* 8:140. 10.3390/sports8110140 33113901 PMC7690681

[B15] Grabow L. Young J. D. Alcock L. R. Quigley P. J. Byrne J. M. Granacher U. (2018). Higher quadriceps roller massage forces do not amplify range-of-motion increases nor impair strength and jump performance. *J. Strength Cond. Res.* 32 3059–3069. 10.1519/JSC.0000000000001906 30152808

[B16] Grabow L. Young J. D. Byrne J. M. Granacher U. Behm D. G. (2017). Unilateral rolling of the foot did not affect non-local range of motion or balance. *J. Sports Sci. Med.* 16 209–218. 28630574 PMC5465983

[B17] Hermens H. J. Freriks B. Disselhorst-Klug C. Rau G. (2000). Development of recommendations for SEMG sensors and sensor placement procedures. *J. Electromyogr. Kinesiol.* 10 361–374. 10.1016/S1050-6411(00)00027-4 11018445

[B18] Hotfiel T. Swoboda B. Krinner S. Grim C. Engelhardt M. Uder M. (2017). Acute effects of lateral thigh foam rolling on arterial tissue perfusion determined by spectral doppler and power doppler ultrasound. *J. Strength Cond. Res.* 31 893–900. 10.1519/JSC.0000000000001641 27749733

[B19] Hirabayashi R. Edama M. Kojima S. Nakamura M. Ito W. Nakamura E. (2018). Effects of reciprocal Ia inhibition on contraction intensity of Co-contraction. *Front. Hum. Neurosci.* 12:527. 10.3389/fnhum.2018.00527 30687045 PMC6336824

[B20] Hirabayashi R. Kojima S. Edama M. Onishi H. (2020). Activation of the supplementary motor areas enhances spinal reciprocal inhibition in healthy individuals. *Brain Sci.* 10:587. 10.3390/brainsci10090587 32847117 PMC7565304

[B21] Kay A. D. Blazevich A. J. (2012). Effect of acute static stretch on maximal muscle performance: a systematic review. *Med. Sci. Sports Exerc.* 44 154–164. 10.1249/MSS.0b013e318225cb27 21659901

[B22] Kay A. D. Husbands-Beasley J. Blazevich A. J. (2015). Effects of contract-relax, static stretching, and isometric contractions on muscle-tendon mechanics. *Med. Sci. Sports Exerc.* 47 2181–2190. 10.1249/MSS.0000000000000632 25668401

[B23] Kelly S. Beardsley C. (2016). Specific and cross-over effects of foam rolling on ankle dorsiflexion range of motion. *Int. J. Sports Phys. Ther.* 11 544–551. 27525179 PMC4970845

[B24] Killen B. S. Zelizney K. L. Ye X. (2019). Crossover effects of unilateral static stretching and foam rolling on contralateral hamstring flexibility and strength. *J. Sport Rehabil.* 28 533–539. 10.1123/jsr.2017-0356 29543123

[B25] Kim S. J. Lee J. H. (2018). Effects of sternocleidomastoid muscle and suboccipital muscle soft tissue release on muscle hardness and pressure pain of the sternocleidomastoid muscle and upper trapezius muscle in smartphone users with latent trigger points. *Medicine (Baltimore)* 97:e12133. 10.1097/MD.0000000000012133 30200103 PMC6133398

[B26] Kiyono R. Onuma R. Yasaka K. Sato S. Yahata K. Nakamura M. (2020a). Effects of 5-week foam rolling intervention on range of motion and muscle stiffness. *J. Strength Cond. Res.* 2020:33044364. 10.1519/JSC.0000000000003757 33044364

[B27] Kiyono R. Sato S. Inaba K. Yahata K. Nakamura M. (2020b). Time course of changes in range of motion, muscle shear elastic modulus, spinal excitability, and muscle temperature during superficial icing. *Sport Sci. Health* 17 341–346. 10.1007/s11332-020-00693-9

[B28] Konrad A. Stafilidis S. Tilp M. (2017). Effects of acute static, ballistic, and PNF stretching exercise on the muscle and tendon tissue properties. *Scand. J. Med. Sci. Sports* 27 1070–1080. 10.1111/sms.12725 27367916 PMC5479471

[B29] Krause F. Wilke J. Niederer D. Vogt L. Banzer W. (2019). Acute effects of foam rolling on passive stiffness, stretch sensation and fascial sliding: A randomized controlled trial. *Hum. Mov. Sci.* 67:102514. 10.1016/j.humov.2019.102514 31499386

[B30] Lacourpaille L. Hug F. Bouillard K. Hogrel J. Y. Nordez A. (2012). Supersonic shear imaging provides a reliable measurement of resting muscle shear elastic modulus. *Physiol. Meas.* 33 N19–N28. 10.1088/0967-3334/33/3/N19 22370174

[B31] Macdonald G. Z. Penney M. D. Mullaley M. E. Cuconato A. L. Drake C. D. Behm D. G. (2013). An acute bout of self-myofascial release increases range of motion without a subsequent decrease in muscle activation or force. *J. Strength Cond. Res.* 27 812–821. 10.1519/JSC.0b013e31825c2bc1 22580977

[B32] Magnusson S. P. Simonsen E. B. Aagaard P. Sørensen H. Kjaer M. (1996). A mechanism for altered flexibility in human skeletal muscle. *J. Physiol.* 497 291–298. 10.1113/jphysiol.1996.sp021768 8951730 PMC1160931

[B33] Morales-Artacho A. J. Lacourpaille L. Guilhem G. (2017). Effects of warm-up on hamstring muscles stiffness: cycling vs foam rolling. *Scand. J. Med. Sci. Sports* 27 1959–1969. 10.1111/sms.12832 28124382

[B34] Morse C. I. Degens H. Seynnes O. R. Maganaris C. N. Jones D. A. (2008). The acute effect of stretching on the passive stiffness of the human gastrocnemius muscle tendon unit. *J. Physiol.* 586 97–106. 10.1113/jphysiol.2007.140434 17884924 PMC2375574

[B35] Naderi A. Rezvani M. H. Degens H. (2020). Foam rolling and muscle and joint proprioception after exercise-induced muscle damage. *J. Athl. Train* 55 58–64. 10.4085/1062-6050-459-18 31855077 PMC6961644

[B36] Nakamura M. Hirabayashi R. Ohya S. Aoki T. Suzuki D. Shimamoto M. (2018). Effect of static stretching with superficial cooling on muscle stiffness. *Sports Med. Int. Open* 2 E142–E147. 10.1055/a-0684-9375 30539131 PMC6259457

[B37] Nakamura M. Onuma R. Kiyono R. Yasaka K. Sato S. Yahata K. (2021a). The acute and prolonged effects of different durations of foam rolling on range of motion, muscle stiffness, and muscle strength. *J. Sports Sci. Med.* 20 62–68. 10.52082/jssm.2021.62 33707988 PMC7919347

[B38] Nakamura M. Sato S. Kiyono R. Yahata K. Yoshida R. Fukaya T. (2021b). Comparison of the acute effects of hold-relax and static stretching among older adults. *Biology (Basel)* 10:126. 10.3390/biology10020126 33562673 PMC7914644

[B39] Nakamura M. Sato S. Kiyono R. Takahashi N. Yoshida T. (2019). Effect of rest duration between static stretching on passive stiffness of medial gastrocnemius muscle in vivo. *J. Sport Rehabil.* 29 578–582. 10.1123/jsr.2018-0376 31094610

[B40] Phillips J. Diggin D. King D. L. Sforzo G. A. (2018). Effect of varying self-myofascial release duration on subsequent athletic performance. *J. Strength Cond. Res.* 35 746–753. 10.1519/JSC.0000000000002751 30024480

[B41] Pickering Rodriguez E. C. Watsford M. L. Bower R. G. Murphy A. J. (2017). The relationship between lower body stiffness and injury incidence in female netballers. *Sports Biomech.* 16 361–373. 10.1080/14763141.2017.1319970 28553879

[B42] Sato S. Kiyono R. Takahashi N. Yoshida T. Takeuchi K. Nakamura M. (2020). The acute and prolonged effects of 20-s static stretching on muscle strength and shear elastic modulus. *PLoS One* 15:e0228583. 10.1371/journal.pone.0228583 32027694 PMC7004320

[B43] Saeki J. Ikezoe T. Yoshimi S. Nakamura M. Ichihashi N. (2019). Menstrual cycle variation and gender difference in muscle stiffness of triceps surae. *Clin. Biomech.* 61 222–226. 10.1016/j.clinbiomech.2018.12.013 30599387

[B44] Smith J. C. Washell B. R. Aini M. F. Brown S. Hall M. C. (2019). Effects of static stretching and foam rolling on ankle dorsiflexion range of motion. *Med. Sci. Sports Exerc.* 51 1752–1758. 10.1249/MSS.0000000000001964 30817716

[B45] Stutzig N. Siebert T. (2017). Assessment of the H-reflex at two contraction levels before and after fatigue. *Scand. J. Med. Sci. Sports* 27 399–407. 10.1111/sms.12663 26887575

[B46] Umehara J. Nakamura M. Saeki J. Tanaka H. Yanase K. Fujita K. (2021). Acute and prolonged effects of stretching on shear modulus of the pectoralis minor muscle. *J. Sports Sci. Med.* 20 17–25. 10.52082/jssm.2021.17 33707982 PMC7919355

[B47] Weppler C. H. Magnusson S. P. (2010). Increasing muscle extensibility: a matter of increasing length or modifying sensation? *Phys. Ther.* 90 438–449. 10.2522/ptj.20090012 20075147

[B48] Wilke J. Müller A. L. Giesche F. Power G. Ahmedi H. Behm D. G. (2020). Acute effects of foam rolling on range of motion in healthy adults: a systematic review with multilevel meta-analysis. *Sports Med.* 50 387–402. 10.1007/s40279-019-01205-7 31628662

[B49] Wilke J. Niemeyer P. Niederer D. Schleip R. Banzer W. (2019). Influence of foam rolling velocity on Knee Range of motion and tissue stiffness: a randomized, controlled crossover trial. *J. Sport Rehabil.* 28 711–715. 10.1123/jsr.2018-0041 29952699

[B50] Witvrouw E. Bellemans J. Lysens R. Danneels L. Cambier D. (2001). Intrinsic risk factors for the development of patellar tendinitis in an athletic population. A two-year prospective study. *Am. J. Sports Med.* 29 190–195. 10.1177/03635465010290021201 11292044

[B51] Yoshimura A. Schleip R. Hirose N. (2020). Effects of self-massage using a foam roller on ankle range of motion and gastrocnemius fascicle length and muscle hardness: A pilot study. *J. Sport Rehabil.* 29 1171–1178. 10.1123/jsr.2019-0281 32050162

[B52] Young J. D. Spence A. J. Behm D. G. (2018). Roller massage decreases spinal excitability to the soleus. *J. Appl. Physiol.* 124 950–959. 10.1152/japplphysiol.00732.2017 29357488

